# The real‐world clinical outcomes and treatment patterns of patients with unresectable locally advanced or metastatic soft tissue sarcoma treated with anlotinib in the post‐ALTER0203 trial era

**DOI:** 10.1002/cam4.4613

**Published:** 2022-02-22

**Authors:** Ren‐Shu Zhang, Jie Liu, Yao‐Tiao Deng, Xin Wu, Yu Jiang

**Affiliations:** ^1^ Department of Medical Oncology, Cancer Center, West China Hospital Sichuan University Chengdu China

**Keywords:** clinical management, nutrition, prognosis, soft tissue sarcoma, target therapy, tyrosine kinase inhibitors

## Abstract

**Background:**

The ALTER0203 clinical trial showed that anlotinib, a multitargeted tyrosine kinase inhibitor, had antitumor effects on advanced soft tissue sarcoma (STS) after the failure of standard chemotherapy. We aimed to evaluate the real‐world efficacy and explore prognostic factors and treatment patterns of anlotinib in patients with advanced STS.

**Methods:**

We retrospectively analyzed the data of patients with unresectable locally advanced or metastatic STS who received at least one dose of anlotinib from June 2018 to March 2021. The survival data were analyzed using the Kaplan–Meier method and compared using the log‐rank test. The Cox proportional hazards model was performed for multivariate analysis.

**Results:**

A total of 209 patients were included. The median age was 48 (range 11–85) years. The median follow‐up, progression‐free survival, and overall survival were 18.7 months, 6.1 months [95% confidence interval (CI): 4.9–7.2], and 16.4 months (95% CI: 13.6–19.1), respectively. The objective response rate was 13.4%. Nutritional status, Eastern Cooperative Oncology Group (ECOG) performance status, and anlotinib treatment patterns (combination therapy or switch maintenance therapy vs. monotherapy) were significantly associated with progression‐free survival. Besides, pathological grade, nutritional status, ECOG performance status, and anlotinib treatment patterns were predictive of overall survival. Due to anlotinib‐related toxicity, 31 (14.8%) patients, and 25 (12.0%) patients experienced dose reduction and treatment discontinuation, respectively.

**Conclusion:**

These findings confirmed the efficacy of anlotinib in patients with advanced STS in a real‐world setting. The patterns of anlotinib treatment deserve further exploration.

## Graphical abstract

Anlotinib is effective in patients with advanced STS in a real‐world setting. FNCLCC grade, ECOG performance status, nutritional status, and anlotinib treatment patterns were predictive of survival in this population and deserve further exploration.

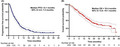

## 1 INTRODUCTION

Soft tissue sarcomas (STS) are a heterogeneous group of rare tumors that account for 0.8–1% of all adult malignancies. STS displays different clinical manifestations and clinical courses with more than 50 different pathological subtypes.^1^ The prognosis for patients with unresectable locally advanced or metastatic STS remains poor. The cornerstone therapy of advanced STS therapy is anthracycline‐based chemotherapy which has a median overall survival of approximately 1 year.^2^, ^3^, ^4^, ^5^ In the past decade, advances in the treatment of unresectable or metastatic STS have included novel chemotherapy agents such as trabectedin and eribulin,^6^, ^7^ highly selective tyrosine kinase inhibitor (TKI) such as crizotinib and imatinib,^8^, ^9^ and multitargeted antiangiogenic TKI such as pazopanib and regorafenib.^10^, ^11^

Another multitargeted TKI, anlotinib exerts inhibitory effects against vascular endothelial growth factor receptors, platelet‐derived growth factor receptors, and fibroblast growth factor receptors.^12^ The phase IIb trial (ALTER0203) was conducted in the Chinese population to explore the antitumor activity of anlotinib in standard chemotherapy‐failed advanced STS, including leiomyosarcoma (LMS), synovial sarcoma (SS), alveolar soft part sarcoma (ASPS), clear cell sarcoma, epithelioid sarcoma, undifferentiated pleomorphic sarcoma, liposarcoma, and fibrosarcoma.^13^ The results showed that the median progression‐free survival (PFS) of the anlotinib group was significantly longer than that of the placebo group (6.27 vs. 1.47 months), and patients with LMS, SS, or ASPS subtypes were more likely to benefit from anlotinib treatment.^13^ Based on these data, anlotinib has been approved in China for the treatment of advanced STS. The international phase III randomized clinical trial (APROMISS) comparing the efficacy of anlotinib and dacarbazine in patients with SS showed that anlotinib improved median PFS (2.89 vs. 1.64 months).^14^ However, both clinical trials strictly selected age, Eastern Cooperative Oncology Group (ECOG) performance status, and pathological subtypes.

Except for pathological subtypes, a study showed that anlotinib treatment‐related adverse events, including increased thyroid stimulating hormone, hypertriglyceridemia, and proteinuria were associated with longer PFS in patients enrolled in the ALTER0203 study.^15^ Patients with longer PFS may have prolonged exposure to drug therapy and may be at higher risk for treatment‐related adverse events. Therefore, the relationship between PFS and treatment‐related adverse events seems to be indistinct.

In this study, we evaluated the real‐world efficacy of anlotinib in patients with advanced STS and explored prognostic factors and treatment patterns in the post‐ALTER0203 trial era.

## 2 METHODS

### 2.1 Study design

This study was a retrospective study approved by the Ethics Committee of Biomedical Research, West China Hospital of Sichuan University. The requirement for individual informed consent was waived. Data were obtained from the Soft Tissue Sarcoma Database of Cancer Center, West China Hospital.

This study included patients who were pathologically diagnosed with STS, had unresectable locally advanced or metastatic STS lesions, and received at least one dose of anlotinib from June 2018 to March 2021. Malignant phyllodes tumor of breast with sarcoma component and dedifferentiated chordoma were also allowed. There were no patients excluded.

The following baseline clinicopathologic data were recorded: age, gender, pathological subtypes, pathological grade according to the French Federation of Cancer Centers Sarcoma Group (FNCLCC) systems,^16^ primary and metastatic sites, largest or symptomatic lesion, tumor size, number of lesions, pretreatment body mass index (BMI), weight change within 6 months before anlotinib therapy, serum albumin and lactate dehydrogenase level, ECOG performance status, comorbidity status, and treatment history. BMI was calculated by body weight (kg) divided by the square of height (m^2^). According to the consensus for cachexia,^17^ malnutrition in the present study was defined as body weight loss >5% in 6 months, weight loss >2% and BMI <20 kg/m^2^, or serum albumin level below the normal lower limit. Switch maintenance therapy referred to patients who achieved disease control after previous chemotherapy were given a sequential treatment of anlotinib until disease progression or unacceptable toxicity. Combination therapy was defined as patients who received other antitumor treatments during anlotinib treatment, including surgery, radiofrequency ablation, radiation, chemotherapy, or immunotherapy.

### 2.2 Treatment

All patients received a once daily oral anlotinib at a dose of 12, 10 mg, or 8 mg based on the patients' conditions. The treatment was administered for 1–14 days in 21‐day cycles. The drug dose of anlotinib was reduced for patients who experienced intolerable adverse events (AEs). Patients continued anlotinib treatment until intolerable AEs or progressive disease (PD) occurred. AEs were assessed using the Common Terminology Criteria for Adverse Events (CTCAE), version 5.0.

### 2.3 Treatment assessment

Treatment response was assessed according to RECIST version 1.1 criteria.^18^ The objective response rate (ORR) was the sum of the rates of confirmed complete response (CR) and partial response (PR). Stable disease should last at least 6 weeks. PFS was calculated from the initiation of anlotinib treatment to disease progression or death from any cause, and overall survival (OS) was calculated from the initiation of anlotinib treatment to death from any cause.

### 2.4 Statistical analysis

Descriptive statistics were used to summarize patient characteristics. For patients lost to follow‐up, the last time of follow‐up was considered the time of death. The cutoff date was August 25, 2021. The Kaplan–Meier method was used to represent the survival curves of PFS and OS, and the log‐rank test was used for comparison. Factors that were statistically associated with survival according to the log‐rank tests were included in the collinearity analysis. Variables with weak collinearity were included in the multivariate Cox proportional regression hazards models to explore independent prognostic factors. A two‐sided *p* < 0.05 was considered statistically significant. The results were described as hazard ratio (HR) with a 95% confidence interval (CI). The statistical software was SPSS (version 17.0), and GraphPad Prism 8.0 was used for plotting.

## 3 RESULTS

### 3.1 Patient characteristics

A total of 209 patients with STS were included in this study, and all of them received anlotinib therapy. The median age was 48 (range 11–85) years, and 106 (50.7%) patients were males. Sixty‐five (31.1%) patients had at least one comorbidity. The most common comorbidity was hypertension (*n* = 25), followed by diabetes (*n* = 12), chronic hepatitis (*n* = 12), and chronic kidney disease (*n* = 9). Nine patients (4.3%) had a history of other malignant tumors including breast cancer (*n* = 3), nasopharyngeal carcinoma (*n* = 2), thyroid cancer (*n* = 1), prostatic cancer (*n* = 1), esophageal cancer (*n* = 1), or diffuse large B cell lymphoma (*n* = 1). The median follow‐up time was 18.7 (range 4.4–38.3) months in living patients.

The most common primary tumor site was the trunk and extremities (43.1%). The majority of patients (83.7%) had one or more metastases, and the most common site was the lung (54.1%). The largest group of pathological subtypes was leiomyosarcoma (18.7%). According to the FNCLCC grading system, 153 (73.2%) cases were grade 2–3. Thirty‐nine (18.7%) patients had an ECOG performance status ≥2. Forty (19.1%) patients had lactate dehydrogenase above the upper limit of normal, and 25 (12.0%) patients with unknown lactate dehydrogenase were classified as normal group. The baseline characteristics are outlined in Table 1.

**TABLE 1:** Baseline characteristics of 209 patients

Characteristics	No. of patients (%)	Characteristics	No. of patients (%)
Age (years)		Lung metastasis	
Median	48	No	96 (45.9)
Range	11–85	Yes	113 (54.1)
<40	80 (38.3)	Bone metastasis	
≥40	129 (61.7)	No	162 (77.5)
Gender		Yes	47 (22.5)
Male	106 (50.7)	Abdominal cavity metastasis	
Female	103 (49.3)	No	170 (81.3)
Pathological type		Yes	39 (18.7)
Leiomyosarcoma	39 (18.7)	Liver metastasis	
Liposarcoma	26 (12.4)	No	172 (82.3)
Dedifferentiated liposarcoma	22 (10.5)	Yes	37 (17.7)
Well‐differentiated liposarcoma	2 (1.0)	Lymph node metastasis	
Pleomorphic liposarcoma	1 (0.5)	No	175 (83.7)
Myxoid liposarcoma	1 (0.5)	Yes	34 (16.3)
Synovial sarcoma	23 (11.0)	Pleural metastasis	
Myxofibrosarcoma	11 (5.3)	No	183 (87.6)
Rhabdomyosarcoma		Yes	26 (12.4)
Alveolar rhabdomyosarcoma	6 (2.9)	Skin, subcutaneous, and muscle tissue metastasis	
Embryonal rhabdomyosarcoma	2 (1.0)	No	191 (91.4)
Pleomorphic rhabdomyosarcoma	2 (1.0)	Yes	18 (8.6)
Anaplastic rhabdomyosarcoma with ALK fusion gene	1 (0.5)	Intracranial metastasis	
Ewing sarcoma	10 (4.8)	No	201 (96.2)
Alveolar soft part sarcoma	9 (4.3)	Yes	8 (3.8)
Epithelioid sarcoma	9 (4.3)	Largest or symptomatic lesion	
Undifferentiated pleomorphic sarcoma	7 (3.3)	Intrapulmonary lesion	61 (29.2)
Malignant peripheral nerve sheath tumor	7 (3.3)	Extrapulmonary lesion	148 (70.8)
Clear cell sarcoma	5 (2.4)	ECOG performance status	
Myoepithelial carcinoma	4 (1.9)	0–1	170 (81.3)
Phyllodes tumor of the breast, malignant	4 (1.9)	≥2	39 (18.7)
Angiosarcoma	4 (1.9)	Co‐morbidities	
Epithelioid hemangioendothelioma	4 (1.9)	No	144 (68.9)
Malignant solitary fibrous tumor	3 (1.4)	Yes	65 (31.1)
Desmoplastic small round cell tumor	3 (1.4)	Lactate dehydrogenase	
Myofibroblastic sarcoma	2 (1.0)	Normal	144 (68.9)
Malignant glomus tumor	2 (1.0)	Above normal upper limit	40 (19.1)
Sclerosing epithelioid fibrosarcoma	1 (0.5)	Unknown	25 (12.0)
Endometrial stromal sarcoma, low grade	1 (0.5)	Serum albumin level	
Malignant granular cell tumor	1 (0.5)	Normal	131 (62.7)
Dedifferentiated chordoma	1 (0.5)	Below normal lower limit	52 (24.9)
Perivascular epithelioid cell tumor (PEComa)	1 (0.5)	Unknown	26 (12.4)
Inflammatory myofibroblastic tumor with ALK fusion gene	1 (0.5)	BMI (kg/m^2^)	
Inflammatory myofibroblastic tumor without ALK fusion gene	1 (0.5)	≤20	53 (25.4)
Aggressive Fibromatosis	1 (0.5)	>20	151 (72.2)
Spindle cell sarcoma, undifferentiated	7 (3.3)	Unknown	5 (2.4)
Round cell sarcoma, undifferentiated	3 (1.4)	Weight change	
Other unclear types	8 (3.8)	Weight gain	17 (8.2)
FNCLCC grade		Unchanged	106 (50.7)
Gx,G1	56 (26.8)	Weight loss	78 (37.3)
G2–G3	153 (73.2)	≤ 5%	26 (12.4)
Driver gene		> 5%	52 (24.9)
Gene fusion	58 (27.8)	Unknown	8 (3.8)
Gene amplification	30 (14.4)	No. of regimens of previous systemic treatment	
Mutation or unknown	121 (57.9)	0	88 (42.1)
Primary site		1	72 (34.4)
Trunk and extremities	90 (43.1)	2	33 (15.8)
Head and neck	21 (10.0)	≥3	16 (7.7)
Thoracoabdominal viscera	46 (22.0)	Targeted therapy history	
Retroperitoneum	34 (16.3)	No	190 (90.9)
Vertebral column, pelvic, and mediastinum	18 (8.6)	Yes	19 (9.1)
No. of lesions		Stratification of ECOG performance status	
1–5	71 (34.0)	ECOG performance status 0–1	
>5	138 (66.0)	Anlotinib monotherapy	82 (39.2)
Largest lesion size (cm)		Anlotinib combination therapy	53 (25.4)
≤5	116 (55.5)	Anlotinib switch maintenance therapy	35 (16.7)
>5	93 (44.5)	ECOG performance status ≥2	
Stage		Anlotinib monotherapy	30 (14.4)
Locally advanced	34 (16.3)	Anlotinib combination therapy	9 (4.3)
Metastatic	175 (83.7)	Anlotinib switch maintenance therapy	0 (0)

Abbreviations: ALK, anaplastic lymphoma kinase; BMI, body mass index; ECOG, Eastern Cooperative Oncology Group; FNCLCC, French Federation of Cancer Centers Sarcoma Group systems.

### 3.2 Clinical outcomes

Before anlotinib treatment, 121 (57.9%) patients had received systemic therapy, and 16 (7.7%) patients had received three or more systemic regimens. During anlotinib treatment, 112 (53.6%) patients received anlotinib monotherapy, 62 (29.7%) patients received anlotinib in combination with other antitumor treatments, and 35 (16.7%) patients received anlotinib as switch maintenance treatment after chemotherapy.

Of 62 cases of anlotinib combination therapy, 26 were combined with systemic therapy including chemotherapy (*n* = 19) and immunotherapy (*n* = 7), and 23 were combined with local therapy including radiotherapy (*n* = 14), radiofrequency ablation (*n* = 2), interventional chemoembolization (*n* = 1), resection of metastatic lesions (*n* = 4), and resection of primary lesions (*n* = 2). The remaining 13 cases were combined with both systemic and local treatment, including chemotherapy combined with radiotherapy (*n* = 6), immunotherapy combined with radiotherapy (*n* = 2), chemotherapy combined with resection of metastatic lesions (*n* = 3), and chemotherapy combined radiotherapy and resection of primary lesion (*n* = 2). The chemotherapy regimens included doxorubicin + ifosfamide (*n* = 5), gemcitabine + docetaxel (*n* = 5), ifosfamide + etoposide (*n* = 3), doxorubicin + cyclophosphamide (*n* = 1), vincristine + doxorubicin + cyclophosphamide (*n* = 1), doxorubicin + cisplatin (*n* = 1), pirarubicin + dacarbazine (*n* = 1), doxorubicin (*n* = 7), epirubicin (*n* = 2), irinotecan (*n* = 2), eribulin (*n* = 1), and capecitabine (*n* = 1). The immunotherapy agents include pembrolizumab (*n* = 4), toripalimab (*n* = 3), and sintilimab (*n* = 2). Of the four patients who underwent resection of primary lesions, three achieved a PR to systemic treatment, and one obtained oncologically appropriate margins (R0). Of the 30 patients who underwent anlotinib combined with chemotherapy, nine (30.0%) patients achieved a PR, 20 patients maintained stable disease (SD), and one had disease progression. Of the nine patients who underwent anlotinib combined with immunotherapy, three (33.3%) patients achieved a PR, five patients remained SD, and one had disease progression. In the anlotinib combination therapy cohort, 15 patients achieved a PR, with an ORR of 24.2%.

Of 35 cases of anlotinib switch maintenance treatment, 30 received first‐line chemotherapy, four received second‐line, and one received three‐line chemotherapy. The median number of chemotherapy cycles administered was six (range 2–21). The chemotherapy regimens included doxorubicin + ifosfamide (*n* = 19), gemcitabine + docetaxel (*n* = 2), ifosfamide + etoposide (*n* = 2), vincristine + actinomycin‐D + cyclophosphamide (*n* = 1), epirubicin + ifosfamide (*n* = 1), doxorubicin monotherapy (*n* = 5), epirubicin monotherapy (*n* = 4), and paclitaxel monotherapy (*n* = 1). At the cutoff time (August 25, 2021), one patient achieved a CR and three patients achieved a PR after anlotinib switch maintenance, with an ORR of 11.4%.

**FIGURE 1:**
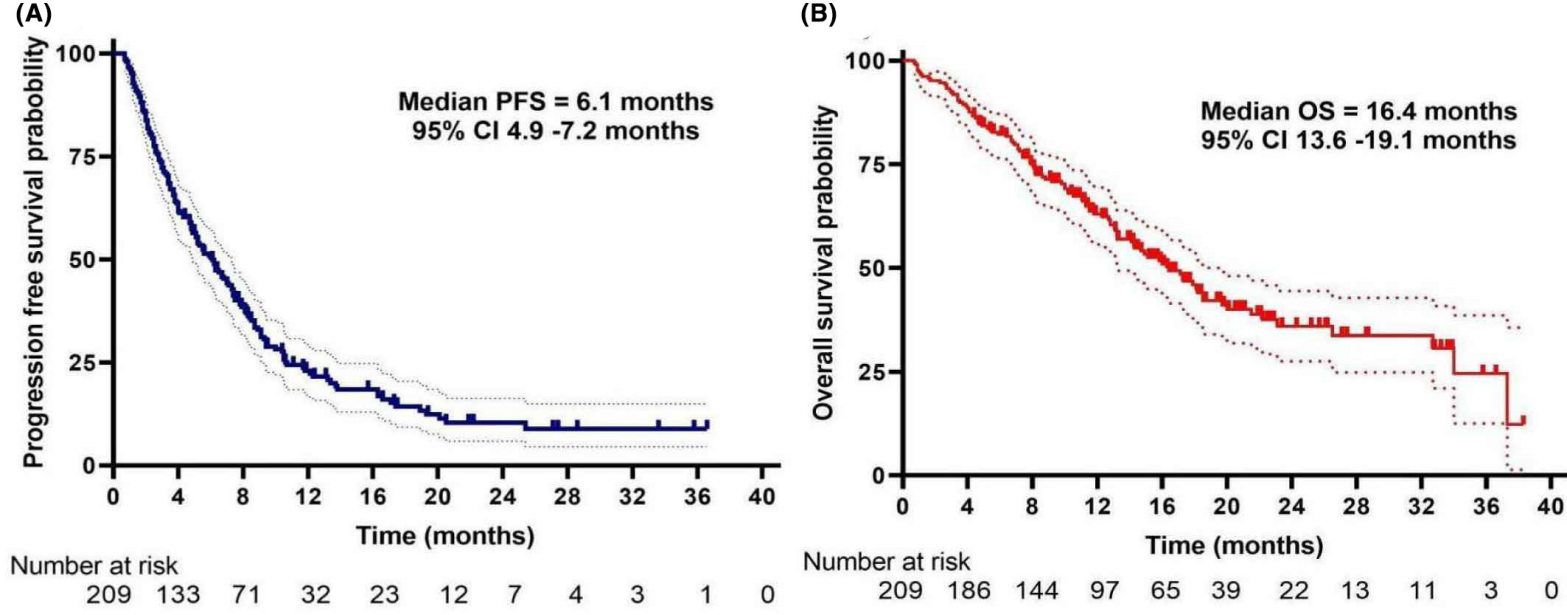
Kaplan–Meier curves of progression‐free survival (A) and overall survival (B) for all patients

Among the 209 patients, two (1.0%) patients achieved a CR and 26 (12.4%) achieved a PR as the best response, with an ORR of 13.4%. The highest ORR was observed in the anlotinib treatment pattern of anlotinib combination therapy (24.2%), followed by switch maintenance therapy (11.4%) and monotherapy (8.0%) (Table 2). The number of PFS events were 168 (80.4%), including 13 (6.2%) deaths. Until the cutoff date, death occurred in 98 (46.9%) patients. Ten (4.8%) patients were lost to follow‐up, which was also considered a death event. Of 101 (48.3%) survivors, 42 patients were still treated with anlotinib. The median PFS and OS were 6.1 months (95% CI: 4.9–7.2) and 16.4 months (95% CI: 13.6–19.1), respectively. Survival curves are shown in Figure 1.

**TABLE 2:** Responses to anlotinib treatment

Subgroups	No. of patients (%)	Tumor response				
		CR No.(%)	PR No.(%)	SD No.(%)	PD No.(%)	ORR (%)
Anlotinib treatment patterns						
Monotherapy	112 (53.6)	1 (0.9)	8 (7.1)	59 (52.7)	44 (39.3)	8.0
Combination therapy	62 (29.7)	0 (0.0)	15 (24.2)	39 (62.9)	8 (12.9)	24.2
Switch maintenance therapy	35 (16.7)	1 (2.8)	3 (8.6)	26 (74.3)	5 (14.3)	11.4
Nutritional status						
Well nourished	124 (59.3)	2 (1.6)	20 (16.1)	82 (66.2)	20 (16.1)	17.7
Malnourished	85 (40.7)	0 (0.0)	6 (7.1)	42 (49.4)	37 (43.5)	7.1
ECOG performance status						
0–1	170 (81.3)	2 (1.2)	26 (15.3)	112 (65.9)	30 (17.6)	16.5
≥2	39 (18.7)	0 (0.0)	0 (0.0)	12 (30.8)	27 (69.2)	0.0
FNCLCC grade						
Gx,G1	56 (26.8)	0 (0.0)	10 (17.9)	34 (60.7)	12 (21.4)	17.9
G2–G3	153 (73.2)	2 (1.3)	16 (10.5)	90 (58.8)	45 (29.4)	11.8
Total	209 (100.0)	2 (1.0)	26 (12.4)	124 (59.3)	57 (27.3)	13.4

Abbreviations: CR, complete response; ECOG, Eastern Cooperative Oncology Group; FNCLCC, French Federation of Cancer Centers Sarcoma Group systems; ORR, objective response rate; PD, progressive disease; PR, partial response; SD, stable disease.

Grade 3 or higher anlotinib‐related AEs occurred in 53 (25.4%) patients. Due to adverse events, 31 (14.8%) and 25 (12.0%) patients experienced dose reduction and treatment discontinuation of anlotinib, respectively. No treatment‐related deaths occurred. These results are presented in Table 3. In addition, 125 (59.8%) patients discontinued anlotinib treatment due to disease progression, 10 (4.8%) due to personal reasons, and 7 (3.3%) due to surgery.

**TABLE 3:** Grade ≥3 adverse events, dosage reduction, and discontinuation of anlotinib

Events	No. of patients (*n* = 209)	Percentage (%)
Grade ≥3 adverse events		
Any adverse events	53	25.4
Hypertension	25	12.0
Infection	6	2.9
Hemorrhage	5	2.4
Neutropenia	5	2.4
Leukopenia	4	1.9
Triglyceride elevation	4	1.9
Anemia	3	1.4
Pneumothorax	3	1.4
Weight loss	3	1.4
Anorexia	3	1.4
Hand‐foot skin reaction	3	1.4
Thrombocytopenia	1	0.5
Aminotransferase elevation	1	0.5
Immunotherapy‐associated toxicity^a^	1	0.5
Adverse events resulted in dosage reduction	31	14.8
Adverse events resulted in discontinuation	25	12.0

^a^ A patient developed grade 3 skin and mucosal toxicities during the treatment of anlotinib combined with immune checkpoint inhibitor.

### 3.3 Nutritional assessment

The mean (±SD) BMI was 22.4 ± 3.5 kg/m^2^. Fifty‐three (25.4%) patients had a BMI ≤20 kg/m^2^. The BMI of five patients was unknown and was replaced with the mean value. Seventy‐eight (37.3%) patients lost weight before anlotinib treatment, of whom 52 (24.9%) lost more than 5% of their body weight. Unknown weight change in eight (3.8%) patients was considered as no weight change. Fifty‐two (24.9%) patients had serum albumin below the lower limit of normal, and 26 (12.4%) patients with unknown albumin were classified as normal group. A total of 85 (40.7%) patients met the criteria for malnutrition.

### 3.4 Prognostic factor analysis

**FIGURE 2:**
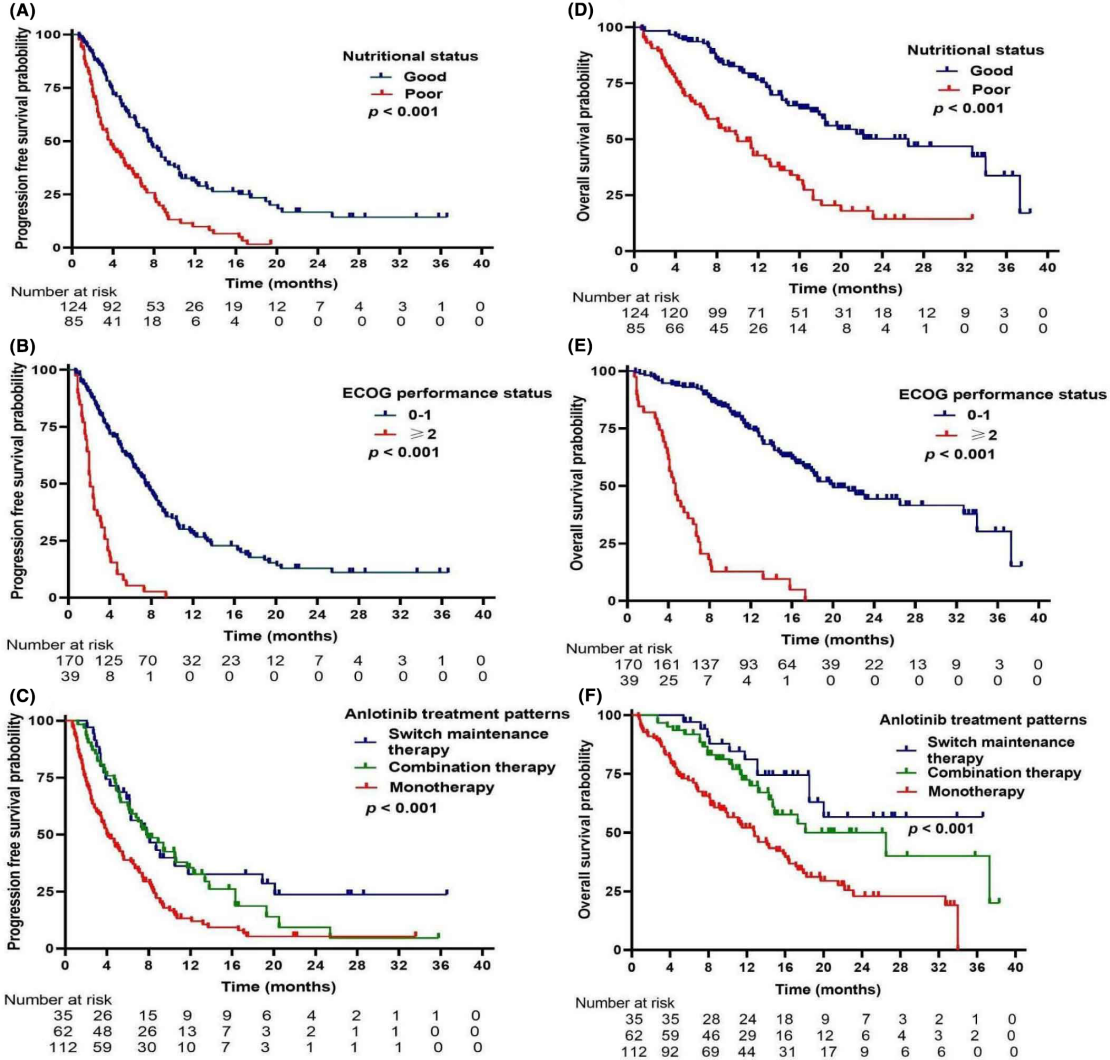
Survival curves of progression‐free survival according to nutritional status (A), ECOG performance status (B), and anlotinib treatment patterns (C). Survival curves of overall survival according to nutritional status (D), ECOG performance status (E), and anlotinib treatment patterns (F)

Baseline characteristics before the time of anlotinib treatment initiation including age, gender, pathological type, FNCLCC grade, primary sites, tumor stage, lesion size >5 cm, largest or symptomatic lesion, number of lesions, nutritional status, serum lactate dehydrogenase, ECOG performance status, driver gene, anlotinib treatment patterns, chemotherapy history, and number of previous systemic treatment regimens were analyzed for the PFS or OS. The log‐rank test showed that anlotinib treatment patterns, serum lactate dehydrogenase, ECOG performance status, nutritional status, lesion size >5 cm, largest or symptomatic lesion, and previous systemic treatment lines were significantly associated with both PFS and OS. Whereas, FNCLCC grade and pathological type were only associated with OS (Table 4). However, there were no differences in age, gender, primary site, tumor stage, number of lesions, driver gene, or chemotherapy history. Survival curves of PFS and OS according to nutritional status, ECOG performance status, and anlotinib treatment patterns are presented in Figure 2. According to collinearity diagnostics, weak collinearity existed among the above variables, which were included in multivariate analysis.

**TABLE 4:** Univariate analysis for PFS and OS

Variables	Median PFS (95%CI)	Log‐rank *p*	Median OS (95%CI)	Log‐rank *p*
Largest or symptomatic lesion				
Intrapulmonary lesion	7.4 (5.0–9.8)	0.015	22.2 (16.6–27.8)	0.020
Extrapulmonary lesion	5.3 (4.2–6.4)		13.2 (10.8–15.6)	
Largest lesion size (cm)				
≤5	7.4 (6.0–8.8)	0.048	22.2 (14.2–30.2)	<0.001
>5	4.8 (3.4–6.2)		12.7 (10.8–14.6)	
Pathological type				
LMS/SS/ASPS	7.0 (3.9–10.1)	0.256	20.0 (9.0–31.0)	0.010
Others	5.9 (4.7–7.1)		13.2 (9.7–16.7)	
FNCLCC grade				
Gx,G1	7.9 (5.5–10.3)	0.153	23.1 (13.0–33.2)	0.005
G2–G3	5.3 (4.3–6.3)		13.2 (10.1–16.3)	
Lactate dehydrogenase				
Normal	6.3 (5.1–7.5)	0.027	18.2 (14.3–22.1)	<0.001
Above normal upper limit	3.8 (2.3–5.3)		11.1 (6.4–15.8)	
ECOG performance status				
0–1	7.5 (6.4–8.6)	<0.001	20.0 (15.8–24.2)	<0.001
≥2	2.1 (1.6–2.6)		4.7 (3.7–5.7)	
Nutritional status				
Well nourished	7.5 (6.0–8.9)	<0.001	26.5 (13.9–39.0)	<0.001
Malnourished	3.8 (2.4–5.1)		10.0 (7.4–12.5)	
No. of regimens of previous systemic treatment				
0	5.5 (3.4–7.6)	0.049	16.3 (13.6–19.0)	0.010
1	6.3 (5.2–7.4)		21.5 (15.1–27.9)	
2	6.3 (5.2–10.0)		16.0 (8.4–23.6)	
≥3	2.8 (0.8–4.8)		8.2 (2.9–13.5)	
Anlotinib treatment patterns				
Monotherapy	4.0 (2.8–5.1)	<0.001	12.8 (9.3–16.2)	<0.001
Combination therapy	7.9 (5.5–10.2)		18.1 (7.3–28.8)	
Switch maintenance therapy	7.8 (4.6–10.9)		Not reach	

Abbreviations: ASPS, alveolar soft part sarcoma; CI, confidence interval; ECOG, Eastern Cooperative Oncology Group; FNCLCC, French Federation of Cancer Centers Sarcoma Group systems; LMS, leiomyosarcoma; OS, overall survival; PFS, progression free survival; SS, synovial sarcoma.

By multivariable Cox repression analysis, independent prognostic factors for PFS were ECOG performance status ≥2 (HR 3.54, 95% CI: 2.20–5.71, *p* < 0.001), malnutrition (HR 1.80, 95% CI: 1.27–2.53, *p* = 0.001), and anlotinib treatment patterns (combination therapy vs. monotherapy; HR 0.62, 95% CI: 0.42–0.89, *p* = 0.010; and switch maintenance therapy vs. monotherapy; HR 0.55, 95% CI: 0.34–0.90, *p* = 0.019; Table 5). The independent prognostic factors for OS were FNCLCC grade G2/G3 (HR 2.48, 95% CI: 1.46–4.21, *p* = 0.001), ECOG performance status ≥2 (HR 5.50, 95% CI: 3.25–9.31, *p* < 0.001), malnutrition (HR 2.01, 95% CI: 1.31–3.08, *p* = 0.001), and anlotinib treatment patterns (combination therapy vs. monotherapy; HR 0.56, 95% CI: 0.34–0.92, *p* = 0.023; and switch maintenance therapy vs. monotherapy; HR 0.41, 95% CI: 0.20–0.81, *p* = 0.011; Table 5).

**TABLE 5:** Multivariate analysis for PFS and OS

Variables	PFS		OS	
	HR (95%CI)	*p*‐value	HR (95%CI)	*p*‐value
With co‐morbidities	1.29 (0.92–1.81)	0.129	1.21 (0.79–1.85)	0.360
Largest or symptomatic lesion (intrapulmonary)	0.80 (0.55–1.16)	0.255	0.83 (0.51–1.34)	0.458
Largest lesion size (>5 cm)	1.03 (0.75–1.42)	0.833	1.48 (0.98–2.23)	0.059
Lactate dehydrogenase (above normal upper limit)	1.04 (0.70–1.55)	0.829	0.98 (0.60–1.58)	0.942
No. of regimens of previous systemic treatment (≥3)	1.07 (0.58–1.97)	0.804	1.38 (0.70–2.70)	0.345
Pathological type (LMS/SS/ASPS)	1.09 (0.77–1.53)	0.612	0.84 (0.53–1.35)	0.488
FNCLCC grade (G2–G3)	1.18 (0.81–1.71)	0.378	2.48 (1.46–4.21)	**0.001**
ECOG performance status (≥2)	3.54 (2.20–5.71)	**<0.001**	5.50 (3.25–9.31)	**<0.001**
Malnutrition	1.80 (1.27–2.53)	**0.001**	2.01 (1.31–3.08)	**0.001**
Anlotinib treatment patterns				
Combination therapy vs. monotherapy	0.62 (0.42–0.89)	**0.010**	0.56 (0.34–0.92)	**0.023**
Switch maintenance therapy vs. monotherapy	0.55 (0.34–0.90)	**0.019**	0.41 (0.20–0.81)	**0.011**

Abbreviations: ASPS, alveolar soft part sarcoma; CI, confidence interval; ECOG, Eastern Cooperative Oncology Group; FNCLCC, French Federation of Cancer Centers Sarcoma Group systems; HR, hazard ratio; LMS, leiomyosarcoma; OS, overall survival; PFS, progression free survival; SS, synovial sarcoma. The bold values means reaching the significance of statistics.

### 3.5 Ad Hoc Analysis by ECOG performance status and treatment patterns

All (35/35) patients receiving anlotinib switch maintenance therapy and most (53/62) patients receiving combination therapy had an ECOG performance status of 0–1. In contrast, most (30/39) patients with an ECOG performance status ≥2 received anlotinib monotherapy (Table 1). This ad hoc analysis explored the survival outcomes of different treatment patterns stratified by ECOG performance status. In patients with a performance status of 0 to 1, anlotinib treatment patterns were still significantly associated with the median PFS (9.4 months for combination therapy vs. 6.5 months for monotherapy, *p* = 0.005; 7.8 months for switch maintenance therapy vs. 6.5 months for monotherapy, *p* = 0.023) and the median OS (26.5 months for combination therapy vs. 16.4 months for monotherapy, *p* = 0.019; not reached for switch maintenance therapy vs. 16.4 months for monotherapy, *p* = 0.020). However, there was no significant difference in survival outcomes among patients with performance status ≥2 who received anlotinib combination therapy or monotherapy (median PFS: 3.1 vs. 2.0 months, *p* = 0.161; median OS: 7.1 vs. 4.1 months, *p* = 0.234).

## 4 DISCUSSION

To the best of our knowledge, this study represented the largest sample size focusing on the clinical outcomes of patients with unresectable locally advanced or metastatic STS treated with anlotinib in the post‐ALTER0203 trial era. The median PFS, median OS, and ORR were 6.1 months, 16.4 months, and 13.4%, respectively (Figure 1). The rates of dose reduction and treatment discontinuation caused by adverse events were 14.8% and 12.0%, respectively.

Multitargeted antiangiogenic therapy is an important treatment in patients with advanced non‐adipocytic STS after the failure of standard chemotherapy. In this population, the median PFS with pazopanib and regorafenib treatment was 4.6 and 4.0 months, respectively.^10^, ^11^ For patients treated with anlotinib, the median PFS was 6.27 months in the ALTER0203 study, which included eight pathological subtypes,^13^ and 2.89 months in the APROMISS study, which focused on one pathological subtype, synovial sarcoma.^14^ Our study included approximately 30 pathological subtypes without selection and exclusion criteria. The median PFS and ORR were 6.1 months and 13.4% for all patients and 4.0 months and 8.0% for patients treated with anlotinib monotherapy, respectively. Only 14.8% of patients had dose reduction, and 12.0% discontinued anlotinib treatment due to adverse events. Our study showed that anlotinib was effective and tolerable in patients with advanced STS in real‐world clinical practice, similar to the above studies.^13^, ^14^

In the ALTER0203 clinical trial, the median PFS with anlotinib treatment was longer in females and patients aged >40 years.^20^ Our study found that neither age nor gender was associated with PFS. One possible reason is the differences in the range of age (18–70 vs. 11–85 years), ECOG performance status (0–1 vs. 0–3), number of pathological subtypes (8 vs. approximately 30), and treatment patterns (monotherapy vs. three patterns) between the ALTER0203 study and our study. However, similar to the finding of the ALTER0203 study that LMS, SS, and ASPS subtypes were more sensitive to anlotinib treatment, our results showed that these three subtypes were associated with longer OS according to univariate analysis (Table 4) but not an independent prognostic factor according to multivariate analysis (Table 5). However, the results of multivariate analysis provided evidence of the prognostic significance of FNCLCC grades in our study (Table 5), which is one of the most commonly used pathological grading systems for STS.^16^

Malnutrition is a severe problem affecting prognosis and quality of life in patients receiving anticancer therapy. Several retrospective studies reported that BMI and weight loss were associated with survival in patients with solid tumors who received TKI therapies.^21^, ^22^, ^23^, ^24^ However, changes in body weight may be masked by weight gain in ascites, peripheral edema, and growth of primary and metastatic tumors.^25^, ^26^ Therefore, in addition to sustained weight loss >5% or weight loss >2% with BMI <20 kg/m^2^, sarcopenia was also taken into consideration in the international consensus for cachexia diagnostic criteria.^17^ Measurement of sarcopenia requires an assessment of muscle mass and strength, of which direct measurement, including cross‐sectional imaging, is preferred. However, there was no clear consensus on screening tools for sarcopenia, and testing methods were complex and inconvenient, making universal measurement difficult for patients.^19^ Several studies have shown that low serum albumin levels are an independent poor prognostic factor in patients with STS and are associated with poor survival.^27^, ^28^, ^29^, ^30^ The serum albumin test is convenient and repeatable. In the present study, a diagnosis of malnutrition was made if patients met one of the following criteria: body weight loss >5% in 6 months, weight loss >2% and BMI <20 kg/m^2^, or serum albumin level below the normal lower limit. These malnutrition criteria might have prognostic power in advanced STS patients treated with anlotinib, as demonstrated by the significant association of malnutrition with survival outcomes (Tables 4 and 5, Figure 2).

The ECOG performance status reflecting the general condition of patients is a common inclusion criterion in clinical trials. Although cancer patients with poor performance status may still benefit from antitumor treatment,^31^ most cancer clinical trials had strict performance status (0 or 1) criteria,^32^ such as the PALETTE, REGOSARC, and ALTER0203 studies.^10^, ^11^, ^13^ Indeed, we found that patients (*n* = 39, 18.7%) with an ECOG performance status of ≥2 had poor survival outcomes (Tables 4 and 5, Figure 2), which was consistent with the findings in patients with advanced non‐small cell lung cancer receiving anlotinib treatment.^33^, ^34^ From the data in Table 1, it was observed that ECOG performance status also generally determined the treatment strategy in clinical practice. Therefore, we conducted an ad hoc analysis to explore the survival difference in treatment patterns stratified by ECOG performance status. Among patients with a performance status of ≥2, there was no significant difference in survival outcomes with combination therapy or anlotinib monotherapy, but in the subset of patients with a performance status of 0 or 1, both anlotinib combination therapy and switch maintenance therapy provided improvements in median PFS and OS compared with anlotinib monotherapy.

Although multitargeted TKI has been an important second‐line treatment option for patients with advanced STS after the failure of standard chemotherapy, the strategies of targeted treatment require further studies. Combination with antiangiogenic TKI and immune checkpoint inhibitor was a promising strategy,^35^, ^36^ but faced the selection of pathological subtype. Another strategy is antiangiogenic TKI in combination with chemotherapy. For 27 patients treated with anlotinib combined with liposomal doxorubicin followed by anlotinib maintenance, the ORR and median PFS were 40.7% and 7 months, respectively.^37^ A recent phase II clinical study reported that the ORR and median PFS were 13.3% and 11.5 months, respectively, in patients treated with epirubicin combined with anlotinib followed by anlotinib maintenance.^38^ Neither study reported OS data due to the duration of follow‐up. The PAPAGEMO phase II trial was conducted in patients with anthracycline‐ and/or ifosfamide‐failed STS and showed that pazopanib combined with gemcitabine significantly prolonged the median PFS compared with pazopanib monotherapy, but with similar median OS.^39^ Our findings suggested that compared with anlotinib monotherapy, anlotinib in combination with other therapies not only improved the ORR and median PFS, but also prolonged the median OS (Tables 4 and 5, Figure 2). In addition to combining chemotherapy, anlotinib was also combined with immunotherapy or local treatment in some cases. This may explain the difference in the findings of the PAPAGEMO study^39^ and our study. Promisingly, switching maintenance therapy with anlotinib after chemotherapy was also significantly associated with longer median PFS and OS (Tables 4 and 5, Figure 2). Possible reasons were that achieving an objective response or stable disease after chemotherapy may have selected patients with good prognosis, and the delayed effects of chemotherapy may add to anlotinib maintenance therapy.^40^ Many trials investigating the strategies of anlotinib treatment in patients with locally advanced or metastatic STS are currently active, such as anlotinib in combination with chemotherapy (NCT03880695, NCT03815474), anlotinib in combination with immune checkpoint inhibitors (NCT04172805, NCT04165330), and switch maintenance therapy with anlotinib after chemotherapy (NCT03890068). However, prospective randomized controlled trials are still lacking.

Several limitations existed in this study. First, there might be selection bias in our data due to the nature of the single‐institutional retrospective study design and the heterogeneity of STS. To reduce selection bias as far as possible, we continuously collected cases from June 2018 to March 2021, and we did not select patients' age, pathological subtypes, or ECOG performance status. Second, the generalizability of our study is unclear because patients in our center mainly came from southwest China. However, the population of Southwest China was over 200 million in 2020. Our center, as a department of a large university‐affiliated tertiary comprehensive hospital, has extensive experience in the management of advanced STS in this region. Third, the sample size of 209 was not very large but had preciousness due to the rarity of STS.

## 5 CONCLUSIONS

Our findings confirmed the antitumor activity of anlotinib in patients with advanced STS in a real‐world setting. The FNCLCC grade, nutritional status, ECOG performance status, and treatment patterns were predictive of survival in this population. The appropriate treatment patterns for anlotinib deserve further exploration.

## CONFLICT OF INTEREST

Yu Jiang has received speakers' honoraria from the Chia Tai Tianqing Pharmaceutical Group Co., Ltd, and is one of the researchers of the ALTER0203 trial which was funded by this company.

## AUTHOR CONTRIBUTIONS

Ren‐Shu Zhang: Data curation, methodology, formal analysis and writing ‐ original draft, and writing ‐ review and editing; Jie Liu: Data curation, methodology, formal analysis and writing ‐ original draft, and writing ‐ review and editing; Yao‐Tiao Deng: Interpretation, formal analysis, writing ‐ review, and editing; Xin Wu: Interpretation, formal analysis, writing ‐ review, and editing; Yu Jiang: Conceptualization, methodology, interpretation, project administration, and writing ‐ review and editing.

## ETHICS STATEMENT

This study was approved by the Ethics Committee of Biomedical Research, West China Hospital of Sichuan University. The requirement for individual informed consent was waived.

## Data Availability

All data are available from the corresponding author.
